# Acute Antibody-Mediated Rejection in Presence of MICA-DSA and Successful Renal Re-Transplant with Negative-MICA Virtual Crossmatch

**DOI:** 10.1371/journal.pone.0127861

**Published:** 2015-05-29

**Authors:** Yingzi Ming, Juan Hu, Qizhi Luo, Xiang Ding, Weiguang Luo, Quan Zhuang, Yizhou Zou

**Affiliations:** 1 Center for Organ Transplantation, the Third Xiangya Hospital of Central South University, Changsha, Hunan, China; 2 HLA Histocompatibility Laboratory, Department of Immunology, Xiangya School of Medicine, Center South University, Changsha, Hunan, China; 3 Department of Urology Surgery, Xiangya Hospital, Center South University, Changsha, Hunan, China; 4 The Cooperative Innovation Center of Engineering and New Products for Developmental Biology of Hunan Province, Changsha, Hunan, China; Leiden University Medical Center, NETHERLANDS

## Abstract

The presence of donor-specific alloantibodies (DSAs) against the MICA antigen results in high risk for antibody-mediated rejection (AMR) of a transplanted kidney, especially in patients receiving a re-transplant. We describe the incidence of acute C4d+ AMR in a patient who had received a first kidney transplant with a zero HLA antigen mismatch. Retrospective analysis of post-transplant T and B cell crossmatches were negative, but a high level of MICA alloantibody was detected in sera collected both before and after transplant. The DSA against the first allograft mismatched MICA*018 was in the recipient. Flow cytometry and cytotoxicity tests with five samples of freshly isolated human umbilical vein endothelial cells demonstrated the alloantibody nature of patient’s MICA-DSA. Prior to the second transplant, a MICA virtual crossmatch and T and B cell crossmatches were used to identify a suitable donor. The patient received a second kidney transplant, and allograft was functioning well at one-year follow-up. Our study indicates that MICA virtual crossmatch is important in selection of a kidney donor if the recipient has been sensitized with MICA antigens.

## Introduction

Although selective immunosuppressive drugs and biologic agents generally alleviate acute rejection in the solid organ transplant patients, treatment of antibody-mediated rejection (AMR) remains challenging [*1*–*3*]. In order to avoid the risk of AMR in kidney transplantation, a prospective complement-dependent cytotoxicity crossmatches (CDC) and/or flow crossmatches (FXM) are performed prior to renal transplant in many centers [*4*]. These experiments can detect alloantibodies against donor HLA-I and II antigens, but neither test detects alloantibodies against MHC class I related chain A (MICA), because the lymphocytes used in the test do not express MICA antigens on their cell surfaces [*5*, *6*]. The polymorphic MICA molecules, which are expressed on the surface of vascular endothelial cells, might be antigenic targets for AMR [*7*]. Zou and his collaborators were the first to detect MICA alloantibodies in 14 patients with failed kidney allografts [*8*]. In a large blinded retrospective renal transplant observational study, pre-transplant MICA antibodies were associated with kidney allograft rejection and shorter graft survival [*9*]. As a well-documented case of acute rejection due to antibodies against MICA has not been reported, screening for MICA antibodies is not used in routine clinic testing prior to renal transplantation. In this report, we present a case of a patient who suffered early aggressive AMR in the presence of donor specific antibodies (DSA) against MICA after the first renal transplant. During donor selection for re-transplant, a donor with a negative MICA virtual crossmatch and negative lymphocyte CDC and FXM crossmatches was selected. The patient received the second kidney transplant, and her allograft was functioning well at one-year follow-up.

## Materials and Methods

### Human samples

Human umbilical cord samples were obtained from the maternity ward at Xiangya Hospital in collaboration with the Department of Obstetrics according to the research plan and protocol approved by Xiangya Hospital Ethnics Committee (EC201403157). Collection of clinic samples for the research was approved by the Ethnics Committee of the 3^rd^ Hospital of Xiangya Medical School (2014-S091). The kidney donor was a 34-year-old man killed in a car accident. Our organ procurement of organization (OPO) obtained the consent from the next kin. All other participants provided written informed consent to participate in this study.

### HLA and MICA typing

Donor typing for HLA-A, HLA-B, and HLA-DR was performed using a PCR-SSP kit (Invitrogen). HLA-A, HLA-B, HLA-C, HLA-DRB1, and HLA-DQB1 typing for the patient and two donors were confirmed using PCR and sequence-specific oligonucleotide probes (PCR-SSOP, Luminex Bead Array, Gen-Probe) according to the manufacturer’s protocol. MICA typing was performed using Sanger sequence-based typing as described previously [*10*].

### Single antigen bead assay for alloantibody analysis

IgG antibodies against HLA class I (A, B, and C) and class II (DR, DQ, and DP) were detected using a single antigen bead array on a Luminex platform (Gen Probe) according to the protocol suggested by the manufacturer. MICA antibody testing was performed on patient serum samples using single antigen beads conjugated with recombinant MICA*001, *002, *004, *007, *008, *009, *012, *016, *017, *018, *019, and *045. This kit was prepared in our laboratory and validated using the reference sera obtained from the 15^th^ International Histocompatibility and Immunogenetics MICA workshop [*11*]. Antibody specificity was based on normalized mean fluorescence intensity (MFI) greater than 2000. DSAs were identified based on the reaction of patient sera to the mismatched antigens for a given donor. Some serum samples were also tested for antibody-C1q binding using a commercially available kit (C1qScreen, One Lambda).

### Endothelial cell isolation, culture, and flow cytometry

Umbilical cord veins were cannulated, washed with phosphate buffered saline (PBS) solution, and treated with 0.2% collagenase (Sigma) at 23°C for 20 min. Endothelial cells were collected and cultured for 3 to 5 days in medium prepared using Endothelial Media BulletKits (Lonza Inc.) at 37°C in a humid atmosphere of 5% CO_2_ in air. Cultured cells were harvested and washed with PBS and used for flow cytometry assays. Cultured HUVECs were used within five passages. The purity of isolated endothelial cells was determined by staining with anti-CD31-PE (BD Biosciences) to facilitate endothelial cell gating. MICA expression on the surface of endothelial cells was detected using MICA-specific monoclonal antibody (mAb) 6B3 as described previously [*9*]. Briefly, 50,000 cells were incubated with mAb 6B3 at 1.0 μg/ml at room temperature for 30 min. Goat anti-mouse IgG conjugated with FITC (Jackson ImmunoResearch Laboratories) was added after three washes, and cells were analyzed using a Gallios flow cytometer (Beckman).

### Flow crossmatch and random endothelial cell crossmatch

A standard flow cytometry crossmatch assay (FXM) was used to analyze the patient’s sera and donor’s T and B lymphocytes prepared from peripheral blood by three-color staining with anti-human CD3-PE, CD19-APC, and IgG-FITC mAbs (BD Biosciences). Retrospective FXM was performed using the donor T and B lymphocytes from donor spleen which had been stored at liquid nitrogen. Approximately 0.5 x 10^6^ cells with or without pronase treatment were incubated at room temperature for 30 min with 25 μl of patient serum diluted 1:4. Random endothelial FXM was performed using freshly isolated human umbilical cord vein endothelial cells (HUVECs). HUVECs were incubated with patient serum which had been treated by pooled normal human platelet absorption. Mouse monoclonal antibodies against human IgG conjugated with FITC (BD Biosciences) was added after three washes. Samples were analyzed using a Beckman Gallios flow cytometer. Cutoff values were set with 50 mean channel shift (MCS) for T cells, 70 MCS for B cells, and 60 MCS for HUVECs. The FXM results in IgG MFI values were converted to MCS values using the following formula: MCS value = 1024 * (log(lgG_MFI) + 1)/4.

### CDC crossmatches

The complement dependent cytotoxicity (CDC) crossmatch assay was used to evaluate cell death in the patient’s serum in the presence of complement. Random HUVEC crossmatches were performed by the modified Amos method (using three washes). Serum was treated using pooled human platelet absorption to exclude anti-HLA antibodies before addition to the endothelial cells. Briefly, 1 μl of negative control, positive control mAb, normal human serum, or patient treated serum was dispensed into individual wells of Terasaky microplates, followed by addition of 1 μl of HUVEC suspension (approximately 5000 cells), and samples were incubated for 1 hour at room temperature. After three washes with PRMI-1640 medium, rabbit complement (One Lambda) was added. After further 30-minute incubation at room temperature, the cells were stained with EB/AO and FluoroQuench (One Lambda) to enable detection of the killing ratio by fluorescence microscopy.

### MICA virtual crossmatch

Since T and B lymphocyte crossmatches do not response to the presence of anti-MICA antibodies, and it is difficult to obtain donor endothelial cells, MICA ‘virtual crossmatch’ was therefore used to detect sensitivity to MICA antigens. We determined whether the patient had MICA-DSA for a given donor by performing MICA genotyping of donor and recipient and testing recipient MICA antibodies using single antigen Luminex flow cytometry. The algorithm of MICA virtual crossmatch was based on the results of missed MICA allele or alleles of a given donor and the specificity of MICA antibodies in recipient’s serum.

## Results

### Clinical case description

**Fig 1:**
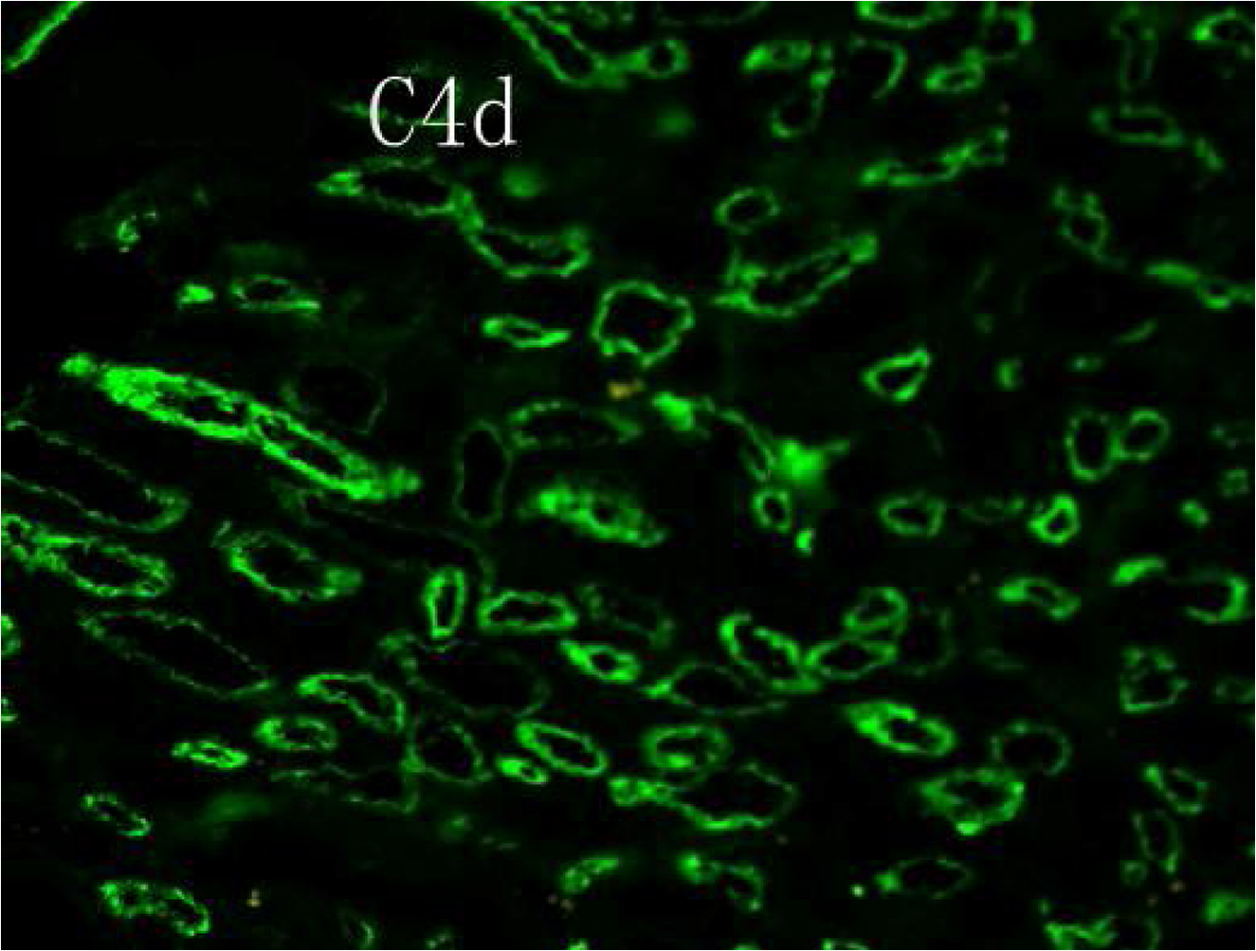
C4d deposition in the walls of glomerular capillaries of allograft. Immunofluorescent microscopic magnification×400.

**Fig 2:**
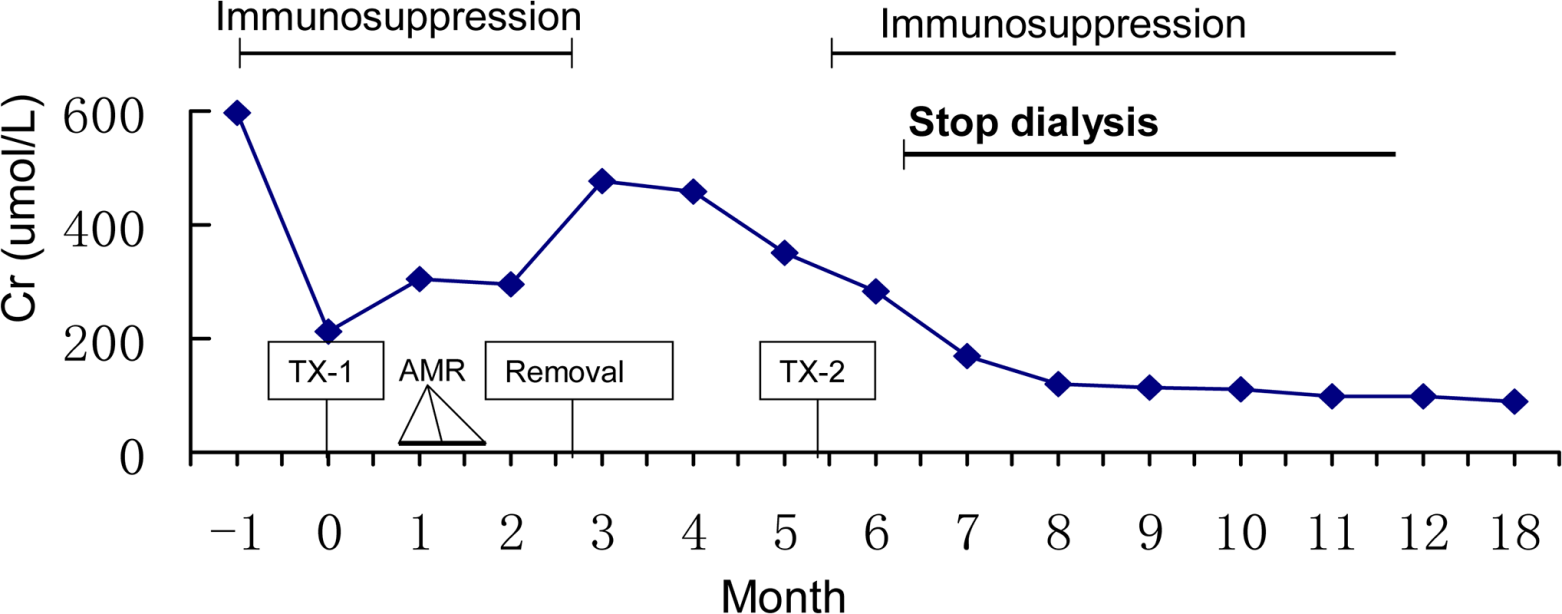
Time line of events and serum creatine levels in the recipient. Serum creatinine levels (Cr, μmol/L) were monitored pre-transplantation, after the first transplant (TX-1) and for one year after the second transplant (TX-2). Times of treatments, AMR diagnosis, and allograft removal are also shown.

The patient is a 48-year-old Han female of blood type A with end-stage renal disease due to glomerulonephritis. She received a kidney transplant from a donor after cardiac death (DCD) in January 2013. Although the patient had HLA-I antibodies (panel reactive antibody, PRA, of 32%) before transplant, the transplant was performed because of the negative CDC and FXM crossmatch with the donor; importantly, the donor was zero-antigen mismatched to the recipient at HLA-A, HLA-B, and HLA-DR loci. The recipient’s serum creatinine levels decreased by 75% relative to pre-transplant levels one week after transplantation, but an acute rejection episode occurred after two weeks. The pathological tissue sections from renal biopsy were positive for C4d (Fig 1) and revealed endothelial cell swelling and hemorrhage. The patient’s serum creatinine levels further increased to the level pre-transplantation. Allograft AMR was not reversed despite treatment including plasmapheresis exchange with albumin and followed by low dose intravenous immunoglobulin (100 mg/kg). The recipient received hemodialysis and the kidney allograft was removed three months after transplantation (Fig 2).

### Retrospective antibody analysis

**Fig 3:**
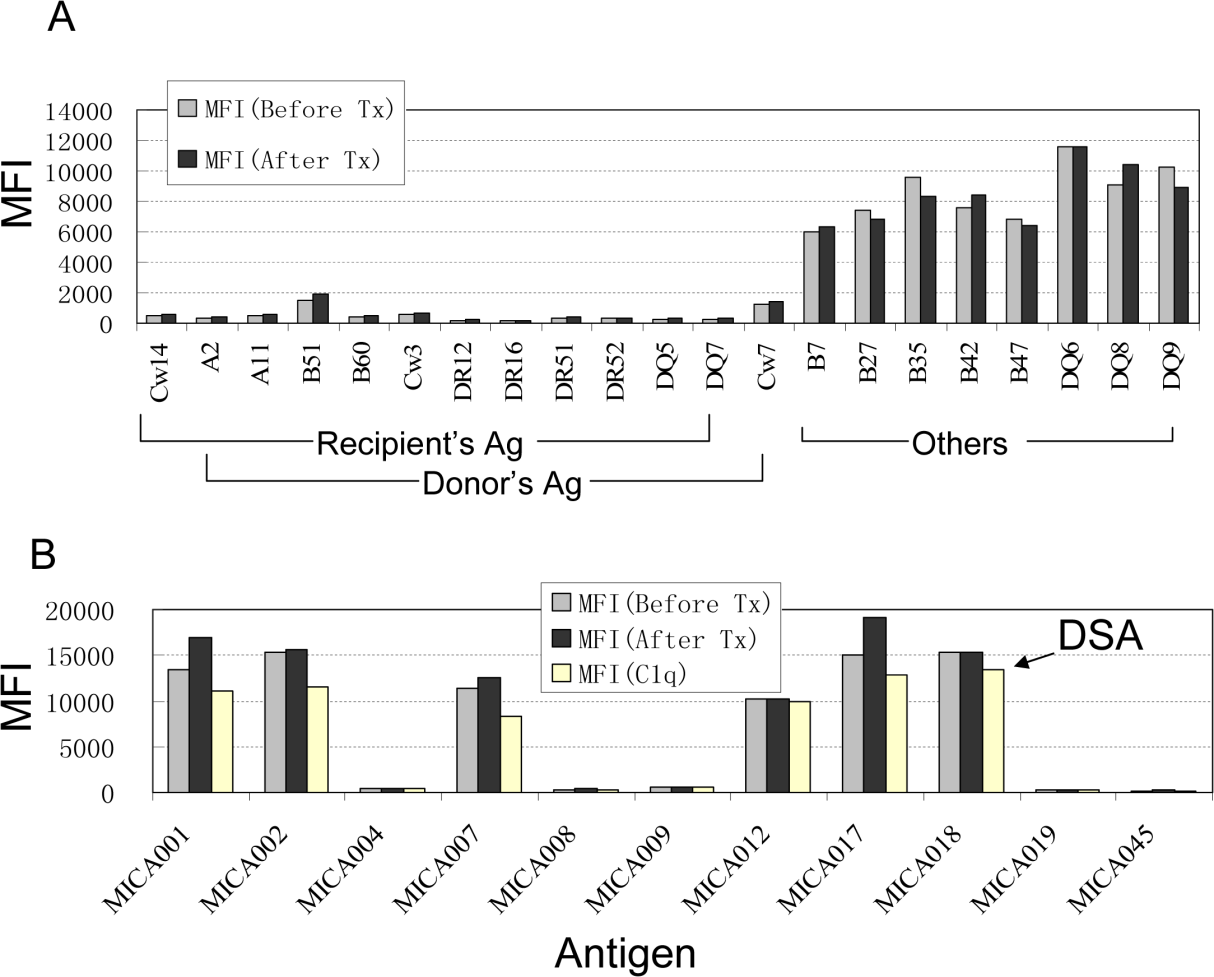
Antibodies against HLA/MICA antigens in pre- and post-transplant serum samples. IgG antibodies against HLA class I and II antigens and MICA antigens were measured by single antigen Luminex beads arrays. MFI values represent the quantitation of alloantibodies detected. (A) The donor’s and recipient’s HLA antigen types and the positive reacted HLA antigens (others) with test serum samples are labeled as grouped.The MFI values of antibodies detected from patient serum before (gray bars) and after the first transplant (solid bar) are given. (B) Anti-MICA antibodies were detected using 11 common MICA antigens. MICA001, MICA002, MICA007, MICA012, MICA017, and MICA018, all from the MICA-G1 antigen group were present in pre- and post-first transplant patient sera; other antigens tested were negative. DSA to the antigen produced by MICA*018 allele is indicated with an arrow. C1q-PE, rather than goat anti human IgG conjugated with PE, was used to demonstrate the complement fix (C1q, white bars).

In order to investigate the possible reason why a zero antigen mismatched kidney allograft failed due to antibody mediated rejection, we tested both pre- and post-transplant serum samples for the presence of HLA-I and II antibodies using the Gen-Probe single antigen Luminex microbeads assay. Both samples had HLA antibodies against antigens of HLA-B7, B64, B65, B27, B2708, B35, B39, B42, B47, B56, B82, DQ6, DQ8, and DQ9. The MFI values were between 2000 and 12000 for these antibodies. There were no significant differences in MFI values of these antibodies before and after transplantation (Fig 3A). Retrospective flow crossmatch was performed between recipient’s serum samples and the donor T and B lymphocytes, which were derived from the donor spleen that had been stored at liquid nitrogen. There were no detectable antibodies in the patient pre- or post-transplant sera that bound donor T or B cells (data not shown). Using MICA single antigen Luminex bead assay, we observed high levels of alloantibodies against MICA group 1 antigen (MICA-G1) in both pre- and post-transplant serum samples (Fig 3B). After normalization, MFI values of antibodies against MICA*001,*002, *007, *012, *017, and *018 antigens were above 10000 and the others were negative. To determine whether the MICA antibodies were donor specific, MICA genotyping was performed using Sanger sequence-based typing of donor and recipient. The recipient’s MICA genotype is MICA*008/MICA*009, and the 1^st^ donor’s genotype is MICA*008/MICA*018 (Table 1). Alloantibodies against MICA*018 antigens were detected in recipient sera; these DSAs likely explain rejection of the first transplant. A C1q binding assay indicated that patient’s MICA antibodies in both pre- and post-transplant serum were complement fixable (Fig 3B). In addition, since two alleles of MICA*008 and MICA*018 were detected in the recipient’s husband DNA sample with MICA genotyping (SBT), the recipient might have been sensitized during her multiple pregnancies prior to transplantation.

**Table 1:** HLA/MICA typing results.

Subject	ABO	HLA-A	HLA-B	Bw	HLA-C	DRB1	DRB3/4/5	DQB1	MICA
Patient	A	A2, A1101	B51, B60	Bw4, Bw6	Cw3, C*14:02	DR12, DR16	DR52, DR51	DQ5, DQ7	*008, *009
Donor 1	A	A2, A1101	B51, B60	Bw4, Bw6	Cw3, Cw7	DR12, DR16	DR52, DR51	DQ5, DQ7	*008, *018
Donor 2	A	A2, A1101	B52, B13	Bw4	Cw3, C*12:02	DR4, DR16	DR53, DR51	DQ5, DQ4	*004, *008

### Random crossmatches to human endothelial cells

**Fig 4:**
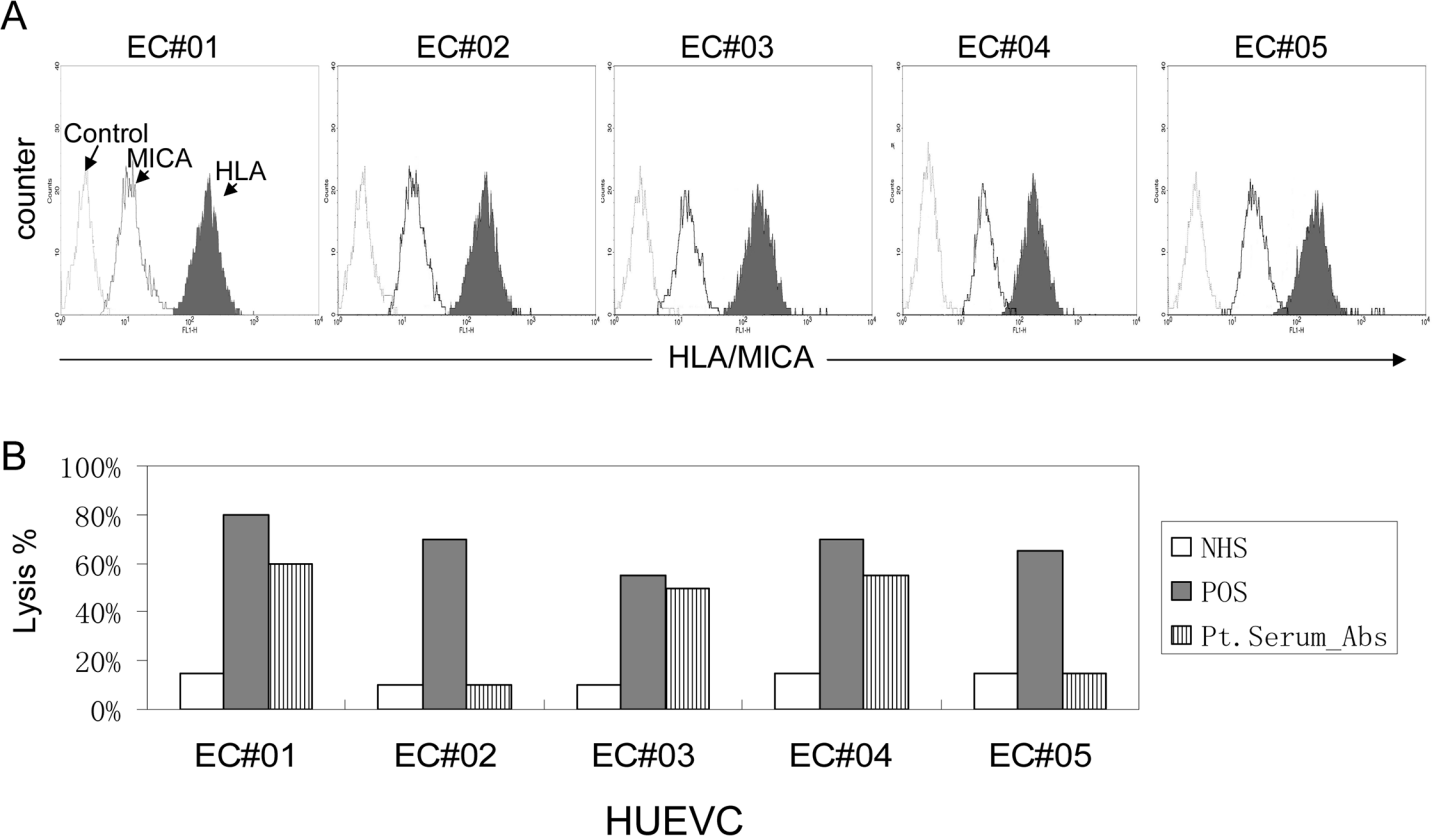
MICA expressed on HUVEC cell surfaces is the target for anti-MICA antibodies. (A) HUVECs were freshly isolated from five umbilical cord samples. Cells were stained with mAb W6/32 for HLA class I antigens (solid profile), 6B3 for MICA antigens (open profile; dark line) and normal mouse IgG as control (open profile; light line). (B). HUVECs were incubated with normal human serum (NHS), pooled PRA+ sera (POS), or patient serum (after first transplant, with platelet absorption). Cytotoxicity is reported as percent lysis.

To further characterize the MICA-DSAs in the recipient’s serum, we evaluated binding to human endothelial cells. Five freshly isolated HUVEC samples were obtained and cultured for 7 days. Each of the samples was positive for staining with anti-MICA-specific monoclonal antibody 6B3 (Fig 4A). In order to exclude HLA class-I alloantibody in recipient serum sample, platelet antibody absorption was employed to remove HLA-class I antibodies from the patient serum. The efficacy of antibody absorption was verified by the HLA-I and MICA single antigen Luminex bead assays. In the treated serum, HLA class I antibodies were present at MFI values less than 1000, but MFI values of MICA antibodies were more than 8000. Random flow crossmatch analyses were performed between HUVEC samples and patient serum (pretreated by antibody absorption and diluted 1:4). HUVECs from donors EC#01, EC#03, and EC#04 were bound by IgG antibodies in tested serum, whereas HUVECs from donors EC#02 and D#05 showed no antibody binding (Table 2). Further, antibodies in patient serum recognized HUVEC samples that expressed membrane molecules encoded by MICA*002, MICA*012, and MICA*018 alleles (Table 2). Further HUVEC CDC crossmatches showed cytotoxicity to these same three HUVEC samples (Fig 4B). Result of in vitro experiments indicated that MICA-DSA in recipient serum had cytotoxicity to cells that expressed MICA-G1 group antigens.

**Table 2:** Random crossmatch between serum and endothelial cells.

Serum^a^	HUEVC MICA genotype^b^				
	EC#01	EC#02	EC#03	EC#04	EC#05
	(002/008)	(008/008)	(008/018)	(009/012)	(004/008)
NHS^c^ (1:4)	0	0	0	0	0
PC^d^ (1:4)	320	286	258	309	267
Pre-serum	178	2	103	192	12
Post-serum	162	36	99	201	39

a: Serum samples were pre-absorbed with pooled human platelets.

b: MICA genotype was determined by Sanger sequence-based typing; MICA alleles are shown in the brackets. The value given is the median channel shift (MCS);

c: NHS (normal human serum) used as negative control, a value of zero indicates MCS background.

d: PC indicates a mixture of pooled MICA-Ab positive sera.

### MICA virtual crossmatch for the secondary kidney transplantation

Six months after her first kidney transplant, the patient was evaluated for a second kidney transplant. The potential donor was blood type A. HLA-B52, HLA-DR4, and HLA-DQ4 antigens were mismatched (Table 1), but recipient did not have HLA antibodies against these mismatched antigens. T and B lymphocyte flow crossmatch was negative as expected. To evaluate MICA histocompatibility, a MICA virtual crossmatch was performed. Donor MICA genotyping showed that MICA*004 was mismatched (Table 1); however, among detectable recipient MICA antibodies, no antibodies responded to the MICA*004 antigen (Fig 3B). These data led us to conclude that the MICA virtual crossmatch was negative. The recipient received the secondary kidney transplant in July 2013, and the kidney allograft was tolerated under the regimen of regular immunosuppressive treatment (Fig 2).

### Patient following up and antibody monitoring

The patient has been followed for one year after the second renal transplant. Measurement of serum creatinine level was performed each week in the first month post-transplantation, then once per month. The serum creatinine levels dropped after the second transplant and have remained with normal levels (<120 umol/L, Fig 2). Single antigen bead assays were used to monitor anti-HLA and anti-MICA antibodies at six and twelve months post-transplantation. No de novo antibody production was observed.

## Discussion

Alloantibodies against human leukocyte antigens (HLA) continue to be the major barrier for the successful renal transplantation. Antibodies against non-HLA antigens such as MICA/B antigens, vimentin [*12*], angiotensin II type 1 receptor[*13*, *14*], tubulin[*15*], myosin[*16*], and collagen[*17*] may also interfere with allograft. Like HLA, MICA antigens are polymorphic and expressed on endothelial cells, dendritic cells, fibroblasts, epithelial cells, and many types of tumor cell; MICA antigens are not expressed on peripheral blood lymphocytes. Sensitization against HLA and MICA can be due to pregnancy, blood transfusions, or previous transplantation.

Patients with high levels anti-HLA antibody wait longer for donor organs than those with lower levels unless a zero antigen mismatched donor is assigned. Even with a zero antigen mismatched donor, some of these patients will have been sensitized to non-HLA antibodies such as MICA antibodies [*18*]. According to the new Banff standard of AMR diagnosis, anti-donor specific alloantibodies (DSA) should be considered prior to transplantation. In the case reported here, the recipient was given a DCD kidney with a zero antigen mismatch, but this allograft was rejected one month after transplantation. Analyses indicated that kidney allograft failed due to AMR, even though there was no HLA-A, B, DR, DQ antigen mismatched to the first donor and negative prospective T and B cell crossmatches.

To determine the cause of the AMR in our patient, we performed a retrospective analysis of MICA genotyping, which revealed potential for MICA-DSAs in the patient. We used pooled normal human platelets to absorb HLA-class I antibodies from patient serum samples as previous described [19], and used this patient sera to perform the random endothelial crossmatches. Our investigation demonstrated that MICA is expressed on human endothelial cell surfaces. The MICA-DSA in recipient serum bound to the cultured HUVECs that expressed MICA-G1 antigens, and the patient’s serum was cytotoxic to these HUVECs, but not to those that did not express MICA-G1, in the presence of complement. This result supports our hypothesis that the failure of the first renal transplant in this patient was due to the presence of MICA-DSAs.

Routine T and B cell crossmatch is not able to detect donor-specific MICA alloantibodies. If a transplant patient has MICA-DSAs, donor kidney endothelial cells become the target of injury. The patient in our study had been sensitized to MICA antigens as well as HLA before transplantation. The patient’s HLA alloantibodies were not specific to the first kidney donor, but the MICA alloantibodies were. The titer of MICA-DSA was very high (MFI > 12,000). Fortunately, the second renal transplant with a negative MICA virtual crossmatch has been successful. In conclusion, a virtual crossmatch for MICA, performed in addition to the HLA histocompatibility assay, will benefit organ transplant recipients.
